# Academy for Multidisciplinary Neurotraumatology – The multidisciplinary pathway towards research and education in neurotraumatology

**DOI:** 10.25122/jml-2022-1008

**Published:** 2022-07

**Authors:** Stefana-Andrada Dobran, Alexandra-Mihaela Gherman, Dafin Fior Muresanu

**Affiliations:** 1. RoNeuro Institute for Neurological Research and Diagnostic, Cluj-Napoca, Romania; 2. Sociology Department, Babes-Bolyai University, Cluj-Napoca, Romania; 3. Department of Neuroscience, Iuliu Hatieganu University of Medicine and Pharmacy, Cluj-Napoca, Romania

The Academy for Multidisciplinary Neurotraumatology (AMN) represents a long**-**standing scientific community with members worldwide. Over the years, the AMN has organized various educational events, the most recent being the Neurotrauma Treatment Simulation Center (NTSC) in Vienna, where participants with diverse backgrounds (neurology, neurosurgery, intensive care, anesthesiology) from 6 countries including Romania, Poland, the Philippines, Mexico, Egypt, and Slovakia participated in a multimodal training following the pathway of the neurotrauma patients through the health system.

The organization is concerned with promoting a new perspective to neurotrauma research and education, one that considers integrative solutions, dynamic collaborations, and innovative approaches. The AMN supports communication among national and international academies, associations, and societies focusing on neurotraumatology in research, education, and practical applications. The society presents a commitment to excellence in education through offering support and encouraging the cooperation of the above-mentioned entities. Furthermore, the AMN can be consulted for expert opinions on matters related to neurotraumatology **–** under special circumstances **–** serving authorities, medical personnel, representative bodies, and third parties at international level.

## THE MULTIDISCIPLINARY APPROACH

Traumatic Brain Injuries (TBI) represent a major issue globally, more direly in low- and middle-income countries [1]. The impact of neurotrauma can be long-term and it can affect the individual on multiple levels, including physical, cognitive, neurobehavioral and emotional. Although certain groups present a higher risk for neurotrauma, it is encountered in populations of any ages and of various demographic characteristics. The need for an integrative approach comes from the wide-range of causes for neurotrauma, from the diverse symptoms secondary to the TBI, as well as from the need of better understanding of neurorehabilitation approaches. For this reason, the AMN is directly involved in the development of patient registries, and entirely committed to offering its support to entities alike concerned with building similar evidence databases. At the same time, as the interest of the patient is of utmost importance, a special attention is given to the constructs enhanced by Artificial Intelligence (AI), so digital applications put in place to record, track, and monitor outpatient data are highly considered.

Neurotrauma research and care have a wide variability at global level, due to the specific needs and obstacles encountered in each country [1]. For this, an “ecological” perspective, encompassing all impact factors such as human factors, health systems, and management approaches are of dire importance for obtaining a true overview of the issue.

Considering all the specialists who have active roles in the patient's pathway, including emergency medicine doctors, neurologists, neurosurgeons, family medicine physicians, psychologists, rehabilitation specialists, physiatrists, kinetotherapeuts, psychiatrists and plenty more, the need for collaborative approaches is increasingly pressing.

Now, with the new technologies and research developed worldwide, it is dire to promote the exchange of information and skills, to better aid the patients, their families, as well as the communities. It is important that not only the specialists, but the large population have a more comprehensive understanding about the impact of neurotrauma.

## AMN - THE LEADING SCIENTIFIC SOCIETY IN NEUROTRAUMATOLOGY

With over 600 members worldwide, AMN represents a leading example for multimodal approaches. With over 8 scientific partnerships and over 45 educational events so far including 9 congresses (starting back in 2008) the AMN promotes education and research through the yearly AMN Congress, where distinguished members of the research community worldwide, with diverse backgrounds, can share their expertise and findings, and debate topics of interest in a multidisciplinary environment.

Moreover, the Academy partners with a wider range of events, including the webinar series “Towards redefining treatment strategies for neurorecovery in stroke and TBI” [2], the “Neurotrauma Treatment Simulation Center (NTSC) Vienna” [3], and numerous other educational events. This science and education portfolio will be further increased via the new set in place considering collaboration of the AMN alongside “World Federation of NeuroRehabilitation” [4], with the “World Health Organization (WHO)” [5], as part of the Academy's advocacy pillar. The interaction with WHO will be multi-levelled, targeting the domains of neurology, brain health, epilepsy and other neurological problems.

## PRESENT (PATIENT REGISTRY – ESSENTIAL NEUROTRAUMA)

When considering research and development, more and more studies are emerging worldwide yearly. Nonetheless, issues regarding the centralization of data for neurotrauma cases remain unresolved in a large number of countries. For this, PRESENT (Patient REgistry – Short Essential NeuroTrauma) [6] aims to cover the gaps and to offer a facile solution that can be implemented in a wide-array of countries. The registry aims to give insight on the real image of neurotrauma and to promote the collection of integrated and diverse variables on neurotraumatology cases. Understanding the prevalence, incidence rates, and the impact on the population, both at an economic and social level, could support the development of better approaches to management and multimodal treatment of neurotrauma and could encourage research both at national and international level.

## THE NEW IMAGE OF AMN

To promote a more dynamic image, the AMN recently launched a new and improved website [7], where one can find information on the upcoming and past events, on the organization itself, and where education in neurotrauma is promoted through the Global Neurotrauma Blog. All specialists interested in improving their knowledge and participating in the development of the domain of neurotraumatology are encouraged to sign up for membership and follow the Academy's monthly newsletter showcasing the latest news on the AMN. Only together, through collaborative efforts, we can improve the knowledge and practices on neurotrauma!
